# *Cichorium intybus* L. promotes intestinal uric acid excretion by modulating ABCG2 in experimental hyperuricemia

**DOI:** 10.1186/s12986-017-0190-6

**Published:** 2017-06-13

**Authors:** Yu Wang, Zhijian Lin, Bing Zhang, Anzheng Nie, Meng Bian

**Affiliations:** Department of Clinical Chinese Pharmacy, School of Chinese Pharmacy, Beijing University of Chinese Medicine, Beijing, China

**Keywords:** *Cichorium intybus* L., Chicory, Hyperuricemia, Intestinal uric acid excretion, ABCG2

## Abstract

**Background:**

Excessive production and/or reduced excretion of uric acid could lead to hyperuricemia, which could be a major cause of disability. Hyperuricemia has received increasing attention in the last few decades due to its global prevalence. *Cichorium intybus* L., commonly known as chicory, is a perennial herb of the asteraceae family. It was previously shown to exert potent hypouricemic effects linked with decreasing uric acid formation in the liver by down-regulating the activity of xanthine oxidase, and increasing uric acid excretion by up-regulating the renal OAT3 mRNA expression. The present study aimed to evaluate its extra-renal excretion and possible molecular mechanism underlying the transporter responsible for intestinal uric acid excretion in vivo.

**Methods:**

Chicory was administered intragastrically to hyperuricemic rats induced by drinking 10% fructose water. The uricosuric effect was evaluated by determining the serum uric acid level as well as the intestinal uric acid excretion by HPLC. The location and expression levels of ATP-binding cassette transporter, sub-family G, member 2 (ABCG2) in jejunum and ileum were analyzed.

**Results:**

The administration of chicory decreased the serum uric acid level significantly and increased the intestinal uric acid excretion obviously in hyperuricemic rats induced by 10% fructose drinking. Staining showed that ABCG2 was expressed in the apical membrane of the epithelium and glands of the jejunum and ileum in rats. Further examination showed that chicory enhanced the mRNA and protein expressions of ABCG2 markedly in a dose-dependent manner in jejunum and ileum.

**Conclusion:**

These findings indicate that chicory increases uric acid excretion by intestines, which may be related to the stimulation of intestinal uric acid excretion via down-regulating the mRNA and protein expressions of ABCG2.

## Background

Uric acid is the final production of purines in humans and avian species since the deficiency in uricase can lead to the elevated uric acid level in serum [1]. Hyperuricemia is characterized by high levels of sustained serum uric acid growth. It is not only one of the metabolic diseases which closely relates diabetes, hypertension, insulin resistance, obesity and other diseases, but also promotes the deposit of urate crystals in tissues arousing several pathological conditions such as acute gouty arthritis, urolithiasis and obstructive uropathy [2, 3]. Recent studies show hyperuricemia has a high incidence and prevalence, and hyperuricemia is thought to be linked with the development of economy and changes of lifestyle [4, 5]. Currently, western medicines which are used to regulating the serum uric acid level, such as allopurinol, benzbromarone, are gradually showing severe adverse reactions [6–8]. However, some clinical and experimental studies show that traditional Chinese medicine has a promising effect on lowering serum uric acid levels. A systematic review of random clinical trials from Li XX showed that Chinese herbal medicine may have clinical effects for functional recovery in patients with gout, and lead to a safe control of serum uric acid levels and inflammation severity [9]. Researches from Ding XQ and Hu QH respectively showed that Wuling san and Simiao pill reduced the serum uric acid level significantly and enhanced urate excretion in hyperuricemic mice induced by potassium oxonate [10, 11]. Accordingly, it must be necessary to observe the therapeutic and preventive effect of traditional Chinese medicine and explore its mechanism on hyperuricemia.

The homeostasis of uric acid levels in the body depends on the balance of its production and excretion. It is commonly accepted that two-thirds of the uric acid is excreted into urine by kidneys, and the remaining third via gut excretion [12, 13]. As a main regulator of uric acid excretion, the kidney plays an important role in uric acid handle, including glomerular filtration, tubular reabsorption and secretion. Report indicated that the primary cause about 90% of hyperuricemia is resulted by the insufficient of renal excretion [14]. Therefore, both treatment researches and hyperuricemia mechanism studies have been widely carried out based on renal excretion. Recent research have found several important transporters involved in uric acid excretion on renal tubular, such as glucose transporter 9(Glut9, also known as SLC2A9) [15], urate transporter 1(URAT1, also known as SLC22A12) [16], organic anion transporter 1(OAT1/NPT, also known as SLC22A6) which is the first identified member of the organic anion transporter family in the Kidney [17], and organic anion transporter 3(OAT3, also known as SLC22A8) [18]. However, intestinal excretion, another important pathway of uric acid flux, has not been studied extensively and its mechanism is still unclear. The genome-wide association studies of serum uric acid identified that ATP-binding cassette transporter, sub-family G, member 2(ABCG2, also known as BCRP) is located in a gout-susceptibility locus on chromosome 4q [19, 20]. ABCG2 is expressed in the small intestine and the liver abundantly, and the impaired ABCG2 function is related to an increase of serum uric acid levels [18]. A recent opinion called “the Remote Sensing and Signaling Hypothesis” suggests that multi-specific SLC and ABCG2 transporters in different tissues are parts of an inter-organ and inter-organismal communication network that balances uric acid levels, metabolites and signaling molecules in the setting of an acute or chronic injury to organs [21]. The following analytical study of statistics from human supported the view that, extra-renal ABCG2 compensates to maintain uric acid homeostasis, most likely in the intestine, as renal function declines [22]. Accordingly, ABCG2 may contribute to the intestinal excretion of uric acid as extra-renal elimination pathway [23].

*Cichorium intybus* L., commonly known as chicory, is a perennial herb of the Asteraceae family. Chicory is well known as a cholagogic and diuretic agent in Uighur folk medicine to improve the appetite, to increase digestion and to cure liver diseases, etc. Reports showed chicory has a broad pharmacological action, including anti-inflammatory, anti-oxidant, antidiabetic, anti-hyperlipidemic, hypoglycemic effects and so on [24]. In previous studies, we found chicory could lower serum uric acid effectively, and the antihyperuricemic effect of chicory mainly associates with the decreasing uric acid formation in the liver by inhibiting the activity of xanthine oxidase, and promoting uric acid excretion by up-regulating the renal OAT3 mRNA expression [25, 26]. The above findings exhibit the potential of chicory to be developed into a therapeutic agent for treatment of hyperuricemia. However, influences of chicory on intestinal excretion of uric acid and mechanisms of chicory-mediated hyporuricemic action have not been completely clarified. Therefore, the present investigation was aimed at evaluating the uricosuric action via intestinal excretion pathway. The potential molecular mechanism of its excretion action was investigated by determining protein and mRNA expression levels of ABCG2 in the small intestine in hyperuricemic rats treated with chicory.

## Methods

### Drugs

Chicory used in the study was authenticated by Professor Yong-Hong Yan (Traditional Chinese Medicine Appraisal Teaching and Research Section of Beijing University of Chinese Medicine). Chicory was grinded into powder and weighed. The powder was extracted with water (1 g:10 ml) by heating to reflux for 1 h twice. Then the solution was concentrated by a rotary evaporator after filtering, and diluted to different volume with purified water [27]. Benzbromarone tablets were obtained from Heumann Pharma GmbH (Germany).

### Experimental animals

Ninety-six male Sprague-Dawley rats of SPF grade, weighting 230-250 g were obtained from Beijing SPF Laboratory Animal Technology co., LTD (Certificate of Quality: SCXK-2011-0004). The animals were maintained in plastic cages with water and food available ad libitum. All animals were housed on a 12 h day-night cycle and the temperature and humidity were kept at 22–24 °C and 50%, respectively. All efforts were made to minimize animal suffering and to keep the number of animals used to a minimum. The study protocol was approved by the Animal Care and Ethics Committee in Beijing University of Chinese Medicine.

### Induction of hyperuricemia and drug treatment

Hyperuricemic rats were generated by giving 10% D-fructose (AMRESCO, USA) in drinking water as previously described [28]. The rats were divided randomly into six groups of 16 rats each, which were CON (the control group was given normal drinking water), MOD (the model group was given 10% fructose in drinking water), BEN (the benzbromarone-treated group was given 10% fructose in drinking water and treated with 20 mg·kg-1·d-1 benzbromarone water solution by intragastric administration, using a ball-tip needle), CHI-H (the high-dosage chicory treated group was given 10% fructose in drinking water and treated with 16.7 g·kg-1·d-1 chicory water solution by intragastric administration, using a ball-tip needle), CHI-M (the middle-dosage chicory treated group was given 10% fructose in drinking water and treated with 13.3 g·kg-1·d-1 chicory water solution by intragastric administration, using a ball-tip needle), CHI-L (the low-dosage chicory treated group was given 10% fructose in drinking water and treated with 6.6 g·kg-1·d-1 chicory water solution by intragastric administration, using a ball-tip needle). During the experiment, the CON group and the MOD group were intragastricly treated with saline.

### Effects of chicory on serum uric acid levels

The hyperuricemic rats were induced by the above method. All drugs were given Intragastric administration once a day. Body weight of rats was recorded every 10 days. Blood samples were taken from rats by cutting the trail tips after a 12 h fasting. Then centrifuge the blood samples at 3500 rpm for 10 min, at 4 °C for plasma separation for later analysis. After a forty-day treatment of the experiment, half of animals in each group were killed and then dissected after 12 h fasting, while the other part of rats in each group were continued to treat with drugs and carried on the intestinal uric acid excretion experiment in a period of 10 days in parallel. Serum uric acid levels were detected according to the uric acid assay kit (BioSino Bio-Technology & Science Inc., China).

### Effects of chicory on intestinal uric acid excretion by HPLC

The analysis of Intestinal uric acid excretion was carried out by the modified method previously described and made an update [29]. Rats were anaesthetized by intraperitoneal injection of pentobarbital sodium and cannulated with polyethylene tubing at the upper duodenum and the middle jejunum to make an intestinal loop at the upper half of the intestine after the overnight fasting. The intestinal contents were removed by injecting saline and air slowly. Then the efflux buffer (saline containing 0.3 mM potassium oxonate) was introduced into the intestinal loop using peristaltic pump at the speed of intestinal tract movement for 2 h. After the set time, the efflux buffer in the loop was collected and the length of the whole small intestine and the intestine loop were measured. The concentration of uric acid was quantified by HPLC. Intestinal uric acid excretion was calculated by the following equation: [Intestinal uric acid excretion] = [Uric acid concentration in the intestinal loop] × [Volume of efflux buffer in the intestine loop] × [Length of the whole small intestine]/[Length of the intestinal loop].

Uric acid levels in the efflux buffer were analyzed by HPLC method by using a reverse phase C18 column(4.6 mm × 250 mm,5 μm,Agilent) on a Shimadzu HPLC system, operated at 30 °C. The mobile phase was composed of 0.2% acetic acid water solution and MeOH system. The samples were filtered by 0.22 μm microfiltration membrane after suitable dilution. Twenty microliter of the treated sample was injected into the column and eluted with the mobile phase at the flow rate of 1 mL/min. The eluate was monitored for absorbance at 288 nm. External standard method of uric acid standard (purity ≥ 99%, Sigma-aldrich, USA) was used in this study.

### Measurement effects of chicory on mRNA expressions of ABCG2 in rats by quantitative real-time polymerase chain reaction (qPCR)

The tissues of jejunum and ileum (100 mg) in rats were homogenized in 1 mL Trizol reagent (Thermo Scientific, USA) to extract total RNA. RNA integrity was evaluated by electrophoresis in 1% agarose gel, and the concentration of total RNA was measured with a trace nucleic acid quantitative instrument. The extracted RNA was reverse-transcribed following the manufacturer^,^ s protocol of RevertAid First Strand cDNA Synthesis Kit (Thermo Scientific, USA). Quantitative real-time PCR was performed using Universal SYBR Green Supermix (Bio-rad) and CFX96 (BIO-RAD) at 95 °C 30s followed by 40 cycles at 95 °C for 5 s, and 60 °C for 30 s. The sense and antisense primers for ABCG2 were designed according to the mRNA sequence (GenBank accession number NM_181381). Amplification of PCR fragments spanning different exons was used to prevent contamination by genomic DNA. The sense primer used was 5′- GGC CTG GAC AAA GTA GCA GA-3′, and the antisense primer was 5′- GTT GTG GGC TCA TCC AGG AA-3′. The sense and antisense primers for GAPDH were according to literature report [24]. The sense primer used was 5′- GGT GGA CCT CAT GGC CTA CA-3′, and the antisense primer was 5′- ATT GTG AGG GAG ATC CTC AGT GT-3′. The ABCG2 mRNA expression values were normalized to GAPDH expression.

### Location of ABCG2 and effects of chicory on ABCG2 protein expressions in rats by immunohistochemistry

Formaldehyde-fixed paraffin-embedded tissues of jejunum and ileum were deparaffinized in xylene and rehydrated. Paraffin sections were pretreated with 10 mM sodium citrate buffer solution (pH 6.0), then cool them to room temperature to unmask antigen. After rinsing by PBS for three times, endogenous peroxidase activity was blocked in dark using 3% hydrogen peroxide for 12.5 min. Before staining, the slides were first incubated with 5% normal goat serum for 25 min. All of above operations were at room temperature. Slides should be washed before the next step. Subsequently, paraffin sections were incubated with a 1:200 dilution of ABCG2 polyclonal antibody (B-25, Santa Cruz, USA) at 4 °C overnight. B-25 was diluted in the antibody diluent buffer.

In this case, after incubation with ABCG2 polyclonal antibody and placed them in the incubator for 60 min to return to 37 °C, slides were incubated for 60 min with polymer auxiliary agent, subsequently incubated for 30 min with HRP-conjugated goat anti-rabbit IgG (ZSGB Biotechnology, China). All above behaviors were acted at 37 °C, and slides should be rinsed with PBS thrice for 5 min before the next step. Color development was achieved with a solution-containing DAB kit (ZSGB Biotechnology, China), incubated for 3 min, and rinsed under running water. After counterstaining with hematoxylin, slides were mounted. For each type of tissue, negative control were included, i.e., by omission of the primary antibody by using with PBS.

All measurements were performed with the automated upright microscope system (Olmpus BX53), and five random images from each section obtained from six rats were randomly captured by high-speed color CCD camera (Olmpus DP72CCD). Imagine Pro-Plus6.0 software was used to analyze pictures. The positive immunostained area in a total area under an image field of each section was calculated.

### Measurement effects of chicory on protein expressions of ABCG2 in rats by western blotting method

After weighing, jejunum and ileum samples from each group were homogenized with ball-grinding mill in radioimmunoprecipitation assay lysis buffer containing phenylmethanesulfonyl fluoride (Solarbio Life Sciences, China) and bathed on ice for 5 min. Then the lysate was centrifuged at 12000 rpm, 4 °C for 10 min to extract total proteins. BCA method (Solarbio Life Sciences, China) was performed to determining the amount of the proteins. The total proteins were diluted at suitable multiple and incubated with 4 × sodium dodecyl sulfate (SDS) loading buffers in the boiling water for 10 min. For western blot analysis, 60 μg of treated sample was separated on a 10% SDS-polyacrylamide gel electrophoresis plate with a 5% stacking gel. Proteins were transferred electrophoretically onto polyvinylidene difluoride (PVDF) membrane (Millipore, Germany) using a blotter (LIUYI Laboratories) at 300 mA for 1.5 h. The membrane was blocked with Tris-buffered saline containing 0.1% Tween-20 (TBST) and 5% skimmed milk powder for 2 h at room temperature. The membrane was incubated with rabbit-anti-ABCG2 antibody (1:400, B-25, Santa Cruz) and mouse-anti-β-actin antibody (1:15,000, 60,008–1, Proteintech, USA) in TBST containing 5% skimmed milk powder overnight at 4 °C. After washing the membranes three times with TBST, immunoreactive bands were detected using HRP conjugated Goat Anti-Rabbit IgG (1:10,000, ZB-2301, ZSGB Biotechnology, China) or Goat Anti-Mouse IgG (1:15,000, SA00001–1, Proteintech, USA) as the secondary antibody in TBST for 1.5 h at room temperature. The proteins were visualized using enhanced chemiluminescence (ECL) reagent (Millipore, Germany). The density of bands was analyzed by Image J and normalized toβ-actin.

### Statistical analysis

The data were expressed as the mean ± S.E. The statistical analysis was performed using an one-way ANOVA followed by the Dunnett’s multiple comparison tests to determine levels of significance by SPSS20.0 software. A *P*-value of *P* < 0.05 was considered statistically significant.

## Results

### Growth performance

Rats were in good status and the body weight was increased steadily throughout the entire experimental period. As showed in Table 1, there was no significant difference in the body weight among the six groups during the experimental period.

**Table 1:** Body weight of rats during experimental days (*n* = 16, g)

Groups	0d	10d	20d	30d	40d
CON	251.31 ± 10.79	423.79 ± 14.79	358.12 ± 14.11	398.38 ± 21.24	422.49 ± 25.14
MOD	248.38 ± 11.02	311.45 ± 19.66	347.19 ± 22.97	387.68 ± 27.10	416.23 ± 32.75
BEN	243.45 ± 10.01	312.36 ± 11.58	344.04 ± 14.44	383.26 ± 18.52	405.41 ± 19.26
CHI-H	248.57 ± 11.19	316.72 ± 23.56	357.91 ± 27.60	397.21 ± 33.74	418.50 ± 40.09
CHI-M	247.94 ± 14.50	308.68 ± 22.28	347.85 ± 26.18	391.55 ± 32.40	412.93 ± 37.68
CHI-L	246.23 ± 12.16	315.35 ± 19.69	356.63 ± 22.61	395.39 ± 25.87	417.62 ± 30.46

### Effects of chicory on serum uric acid levels

The 10% fructose-drinking caused a significant increase in serum uric acid levels, and this enhancement could be maintained for 40d steadily (Table 2). As showed in Table 2, chicory given intragastrically at the high and the middle doses once a day for 10d could attenuated serum uric acid levels significantly, moreover the reduced serum uric acid levels could be kept effectively during the whole experiment in fructose-induced hyperuricemic rats when compared to the untreated hyperuricemic rats (*P* < 0.05). The low dose of chicory reduced serum uric acid levels markedly after administrating for 20d (*P* < 0.05), but did not show significant differences during the subsequent experimental days when compared with the untreated hyperuricemic rats. The onset of lowering serum uric acid levels in the benzbromarone group was from the 20th day to the 30th day (*P* < 0.01, *P* < 0.05) when compared with the untreated model group in this experiment.

**Table 2:** Uric acid-lowering effects of intragastric chicory in the hyperuricemic rats (*n* = 16, μmol/L)

Groups	0d	10d	20d	30d	40d
CON	51.47 ± 21.30	66.44 ± 26.12	76.22 ± 22.57	133.80 ± 33.23	74.10 ± 24.41
MOD	56.72 ± 28.76	87.63 ± 27.34^*^	98.07 ± 22.23^**^	177.08 ± 44.99^**^	95.80 ± 18.01^**^
BEN	50.56 ± 26.81	74.66 ± 35.29	75.64 ± 14.69^##^	145.61 ± 36.58^#^	82.60 ± 19.76
CHI-H	50.33 ± 21.91	52.40 ± 16.77^##^	66.17 ± 21.09^##^	145.61 ± 36.58^#^	73.82 ± 35.90^#^
CHI-M	53.08 ± 25.51	55.90 ± 29.62^##^	68.82 ± 16.84^##^	112.52 ± 45.48^##^	83.07 ± 41.07
CHI-L	50.22 ± 21.67	70.05 ± 32.18	65.09 ± 28.36^##^	155.27 ± 44.47	98.11 ± 9.46

Note:^*^ *P* < 0.05, ^**^ *P* < 0.01 vs. the CON group; ^#^ *P* < 0.05, ^##^ *P* < 0.01 vs. the MOD group. Means with different superscript lowercase letters in the same column are significantly different

### Effects of chicory on intestinal uric acid excretion by HPLC

**Fig. 1:**
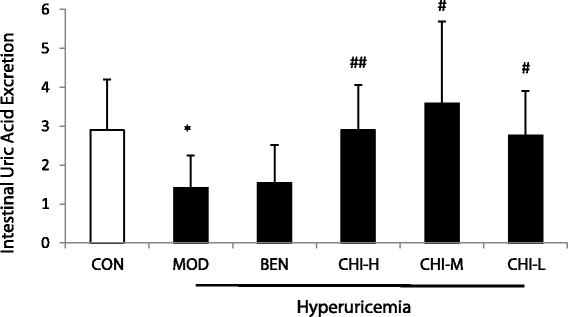
Uricosuric Effects in intestines of Chicory in Hyperuricemic Rats. Data were expressed as mean ± S.E. for six rats in each group. ^*^ *P* < 0.05 vs. CON group, ^#^ *p* < 0.05, ^##^ *P* < 0.01 vs. MOD group

As shown in Table 2 and Fig. 1, 10% fructose-drinking increased uric acid levels in serum significantly as well as decreased the excretion of intestinal uric acid obviously in rats, when compared with the normal control group (*P* < 0.05). Administration of chicory reduced serum uric acid levels significantly in a dose-dependent manner to some degree and enhanced the intestinal uric acid excretion at the high dose(*P* < 0.01), middle dose and low dose(*P* < 0.05) compared with the model group exhibiting the promotion of uric acid excretion in gut. As a positive control, benzbromarone showed a remarkable reduction of serum uric acid but there was no significant change of uric acid excretion of the intestine compared with hyperuricemic rats.

### Effects of chicory on ABCG2 mRNA expressions in jejunum and ileum

**Fig. 2:**
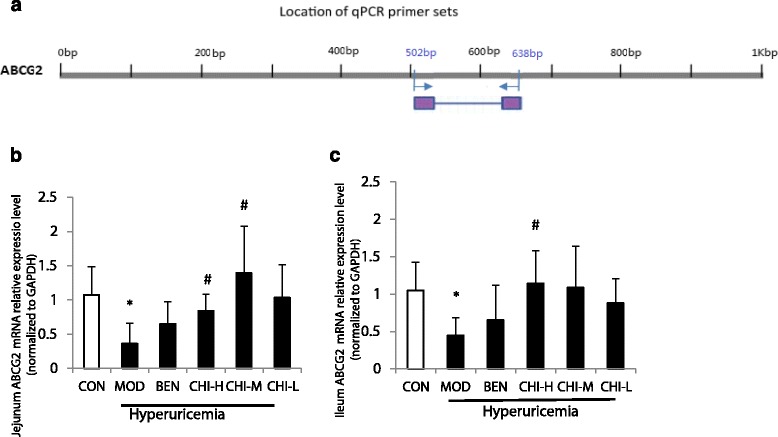
Effects of Chicory on ABCG2 mRNA Expressions. **a** Illustration of the location of primer sets for qPCR amplification of ABCG2 mRNA transcripts. Numbers refer to the translation start site of ABCG2. **b** In jejunum. **c** In ileum. Data were expressed as mean ± S.E. for six rats in each group. ^*^ *P* < 0.05 vs. CON group, ^#^ *p* < 0.05 vs. MOD group

Effects of chicory and benzbromarone on ABCG2 mRNA expressions of jejunum and ileum are shown in Fig. 2, from which it can be seen that fructose-induced hyperuricemic rats showed the remarkable down-regulation of ABCG2 mRNA expressions in jejunum and ileum (*P* < 0.05, *P* < 0.05), when compared with the normal controlled rats. Chicory up-regulated the expression of ABCG2 mRNA was shown in a dose-dependent manner. The high dose of chicory significantly increased ABCG2 mRNA expressions in jejunum (*P* < 0.05) as well as ileum (*P* < 0.05), when compared with the model group. The middle dose of chicory markedly up-regulated ABCG2 mRNA expressions in jejunum (*P* < 0.05), but had no significant change in ileum, when compared with the model group. There were no remarkable difference in the expression of ABCG2 mRNA between the low dose of chicory group and the model group in jejunum or ileum. Similarly, there was no change of ABCG2 mRNA expressions in jejunum still ileum was observed in between benzbromarone and hyperuricemic rats.

### Location of ABCG2 and effects of chicory on ABCG2 protein expressions in jejunum and ileum in rats by immunohistochemistry

**Fig. 3:**
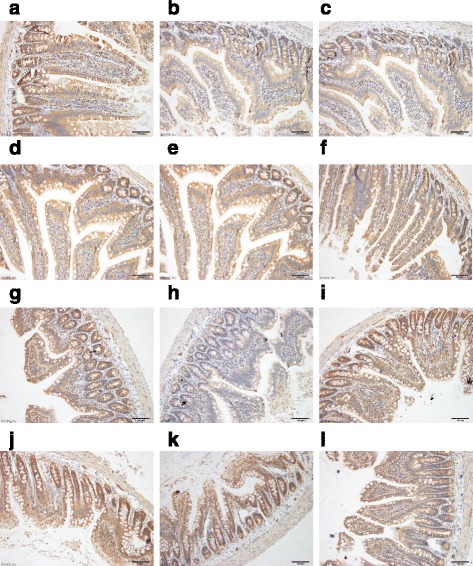
Location of ABCG2 and effects of chicory on ABCG2 protein expression. **a** Jejunum of normal rat ABCG2 IHC stain (×20 objective lens). **b** Jejunum of model rat ABCG2 IHC stain (×20 objective lens). **c** Jejunum of benzbromarone rat ABCG2 IHC stain (×20 objective lens). **d** Jejunum of chicory extract high-dose rat ABCG2 IHC stain (×20 objective lens). **e** Jejunum of chicory extract middle-dose rat ABCG2 IHC stain (×20 objective lens). **f** Jejunum of chicory extract low-dose rat ABCG2 IHC stain (×20 objective lens). **g** Ileum of normal rat ABCG2 IHC stain (×20 objective lens). **h** Ileum of model rat ABCG2 IHC stain (×20 objective lens). **i** Ileum of benzbromarone rat ABCG2 IHC stain (×20 objective lens). **j** Ileum of chicory extract high-dose rat ABCG2 IHC stain (×20 objective lens). **k** Ileum of chicory extract middle-dose rat ABCG2 IHC stain (×20 objective lens). **l** Ileum of chicory extract low-dose rat ABCG2 IHC stain (×20 objective lens). Low expression of model group in jejunum and ileum (*P* < 0.05, *P* < 0.01 vs. control group slices), which developed heavy stains; inhibition of ABCG2 by chicory (*P* < 0 .01, *P* < 0 .01 vs. model group slices)

There was no immunoreactivity in the sections set aside for negative control and ABCG2 positivity was indicated by cytoplasmic golden brown staining. As shown in Fig. 3, prominent staining was observed in the small intestine, with strong apical staining of the epithelium and glands of the jejunum and ileum. From the Table 3, ABCG2 protein expressions of the model group were reduced significantly in the jejunum and ileum (*P* < 0.05, *P* < 0.01), when compared to the control group. An evaluation of ABCG2 protein expressions in all chicory-treated groups was notably higher in the jejunum and ileum compared with the model group (*P* < 0.01), whereas the benzbromarone-treated group showed pronounced increase only in the ileum (*P* < 0.01).

**Table 3:** Effects of Chicory on the ABCG2 protein Expressions in jejunum and ileum by immunohistochemistry (*n* = 6)

	Jejunum		Ileum	
Groups	Positive area (10^5^)	Accumulated integrated optical density(10^4^)	Positive area (10^5^)	Accumulated integrated optical density(10^4^)
CON	4.68 ± 2.11	2.16 ± 1.44	12.56 ± 0.66	4.24 ± 0.49
MOD	3.41 ± 1.77^*^	1.31 ± 0.81^*^	12.90 ± 0.83	3.72 ± 0.77^**^
BEN	4.17 ± 1.70	1.82 ± 1.12	12.82 ± 0.60	4.49 ± 0.65^##^
CHI-H	4.77 ± 1.27^##^	2.00 ± 0.48^##^	13.82 ± 0.29^**##^	8,87 ± 2.03^**##^
CHI-M	5.27 ± 1.02^##^	2.12 ± 0.49^##^	13.82 ± 0.42^**##^	7.46 ± 0.51^**##^
CHI-L	4.26 ± 1.40^#^	1.87 ± 0.84^#^	13.88 ± 0.18^**##^	7.54 ± 0.62^**##^

Note:^*^ *P* < 0.05, ^**^ *P* < 0.01 vs. CON group; ^#^ *P* < 0.05, ^##^ *P* < 0.01 vs. MOD group. Means with different superscript lowercase letters in the same column are significantly different

### Effects of chicory on ABCG2 protein expressions in jejunum and ileum in rats by western blotting

**Fig. 4:**
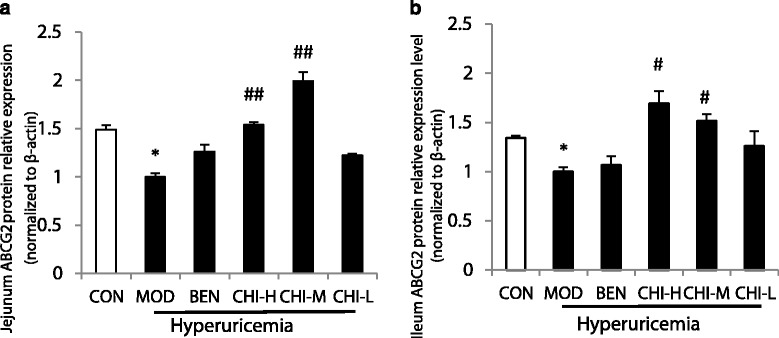
Effects of Chicory on the ABCG2 protein Expressions by western blotting. **a** In jejunum. **b** In ileum. Data were expressed as mean ± S.E. for six rats in each group. ^*^ *P* < 0.05 vs. CON group, ^#^ *p* < 0.05, ^##^ *P* < 0.01 vs. MOD group

**Fig. 5:**
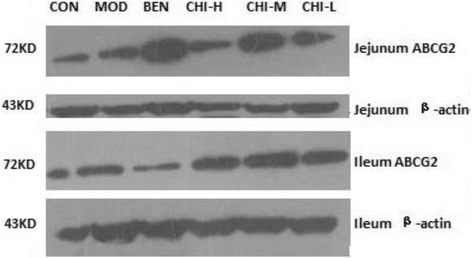
ABCG2 protein Expressions in jejunum and ileum by western blotting

Effects of chicory and benzbromarone on protein expressions of ABCG2 in hyperuricemic rats are shown in Figs. 4 and 5, from which it can be seen that fructose-induced hyperuricemic rats showed the remarkable reduction of ABCG2 protein expressions in jejunum and ileum (*P* < 0.05, *P* < 0.05), when compared with the normal control rats. It also showed that the protein expression levels of each treatment group were elevated in varying degrees in jejunum and ileum by comparing with the hyperuricemic rats. Chicory could increase ABCG2 protein expression, as well as in the ileum (*P* < 0.01, *P* < 0.01), compared with the model group. However, there was no obvious alteration between the low dose of chicory group and the model group in jejunum and ileum. Similarly, there was no change of the ABCG2 protein expression in jejunum still ileum was observed between benzbromarone and hyperuricemic rats.

## Discussion

### Pathomechanism of hyperuricemia induced by fructose

Fructose is widely used in the food industry for its flavor [30]. However, with the growth of fructose consumption, we also witnessed its negative influences. There are more and more speculations that excessive consumption of fructose could cause insulin resistance, obesity, and have a strong correlation with hyperuricemia [31, 32]. A rapid and sustained enhancement of serum uric acid levels in humans will happen after the oral or intravenous ingestion of fructose, especially in gout patients [33, 34]. Recent animal experiments proved that sustained or excessive intake of fructose could cause the abnormal serum uric acid levels [35]. As expected, our studies showed that the drinking of fructose with the concentration of 10% for 10d induced the elevation of serum uric acid levels markedly and maintained steadily in rats during the whole experiment (Table 2). The mechanism of induction of hyperuricemia by fructose is controversial. It is commonly accepted that the cause of hyperuricemia induced by fructose is associated with the formation of urate [36, 37]. In addition, Abdulla, M.H., et al., found that the high dose of fructose intake altered renal haemodynamic and excretory function [38]. Our former study mentioned previously also showed the high dose of fructose had an effect on renal dysfunction. Nevertheless, the change of intestinal uric acid excretion in fructose-induced hyperuricemia is still unclear.

### The role of the intestine in hyperuricemia

Hyperuricemia is traditionally classified into the ‘overproduction’ type, the ‘underexcretion’ type, and the ‘combined’ type. This classification is solely based on the amount of liver uric acid formation and renal urate excretion, and the extra-renal excretion such as gut excretion is not taken into consideration. The process of uric acid handling in the liver and kidneys has been carried out widely, while the mechanism of uric acid elimination in the intestine is poorly understood. Matsuo, H., et al. suggested the ‘overproduction’ type in the current concept of hyperuricemia could be renamed ‘renal overload’ type, which consists of ‘extra-renal urate underexcretion’ and genuine ‘urate overproduction’ type [29]. Their research provided a new concept which is valuable for the understanding of hyperuricemia and emphasized the decreased intestinal urate excretion is a common cause of hyperuricemia. In our study, the intestinal uric acid excretion reduced significantly in hyperuricemia rats when compared with the normal group (Fig. 1). This illustrated that the mechanism of hyperuricemia induced by fructose would be associated with excretion of uric acid in the intestine.

### The medical function of chicory on hyperuricemia

Chicory is widely used as a leafy vegetable, forage grass, raw materials of sugar and coffee substitute materials and it has been a Uighur and Mongolian folk medicine for many years. Its main pharmacological effects include hypoglycemic action, antihyperlipidemic action, hypouricemic action, hepatoprotective effect and the adjustive effect of the digestive system and cardiovascular system [39]. All above indicate that chicory is potential and exploitable in healthy beverages, functional foods and miracle drugs. In the previous study, chicory could significantly decrease serum uric acid levels by inhibiting the activities of uricopoiesis metabolic enzymes of 5′-nucleotidase, adenosine deaminase, purine nucleoside phosphorylase, guanine deaminase and xanthine oxidase. This is an indication that chicory could decrease the formation of uric acid in the liver [40]. In addition, our research also displayed that chicory could lower the serum uric acid levels obviously by up-regulating the renal OAT3 mRNA expression, exhibiting that the hyporicemic effects of chicory could be associated with promoting excretion of uric acid in the kidney [25]. These findings suggested that the hypouricemic action of chicory might be related not only to the inhibition of uric acid formation in the liver, but also the enhancement of renal uric acid excretion. The potent hypouricemic action of chicory in vivo could be explained by the dual actions of production and excretion. In the current study, the high and middle doses of chicory were reduced serum uric acid levels significantly in fructose-induced hyperuricemia rats (Table 2). The uricosuric action of chicory was earlier and more stable than benzbromarone. To further determine if the uricosuric effect observed is linked with the excretion of uric acid in the intestine, we investigated the effect of chicory on the intestinal uric acid excretion in hyperuricemia rats induced by fructose consumption. Administration of chicory once a day for 10d could significantly decrease serum uric acid levels and increase the intestinal uric acid excretion at the high and middle dosages in hyperuricemia rats (Fig. 1). Those results illustrated that chicory has the ability of accelerating intestinal uric acid excretion and is a potent uricosuric agent.

### The role of ABCG2 in hyperuricemia and the regulatory effect by chicory

ABCG2 is a member of the ATP-binding cassette transporter superfamily and has only one ATP-binding cassette and six putative transmembrane domains. This suggests that ABCG2 is a half-transporter, which may function as a homodimer or heterodimer. Recent research showed that the elevated serum uric acid level is associated with decreased ABCG2 activity which is resulted by the genetic polymorphisms [41]. A genome-wide association study (GWAS) of serum uric acid levels proved that ABCG2 has multiple SNPs in a genomic region on chromosome 4 as associated with urate levels and prevalence of gout [42]. ABCG2 is expressed on the apical membrane in several tissues, including liver, intestines and kidneys [43], but the expression of ABCG2 is much higher in the intestinal epithelial cells and hepatocytes than in proximal tubular cell [44]. It has been shown to transport a wide range of substrates, such as nucleoside analogue drug lamivudine, which are structurally similar to urate, fluorochrome rhodamine, folic acid etc. [45]. There is study shown that the ABCG2 is a high-capacity urate secretion transporter [41]. Kolz, M., et al.*,* conducted a meta-analysis of genome-wide association scans from 14 studies totaling 28,141 participants of European descent and the result indicated ABCG2 is closely related to the concentration of serum uric acid [46]. Hosomi A., et al.*,* found that the expression of rABCG2 mRNA was well correlated with uric acid secretory activity into the intestinal lumen [23]. Yano, H., et al., found the urine UA excretion and UA clearance were decreased significantly in a 5/6 nephrectomy rat, whereas the serum uric acid level was not significant, and the expression of ABCG2 in the ileum of the nephrectomy group showed significant upregulation [47]. This suggests that an excretory pathway other than the kidney, probably the intestine, may operate in a complementary role. From the above, ABCG2 would be critical to accelerating intestinal uric acid excretion and has potential to lower serum uric acid.

In the current study, significant staining was observed in jejunum and ileum with strong apical staining of the epithelium as well as gland (Fig. 3). From the analysis of immunohistochemical figures (Table 3), we observed that ABCG2 protein expressions in jejunum and ileum were prominently decreased in the rat model obtained the fructose drinking. The significant promoted ABCG2 protein expression levels by chicory were observed in jejunum and ileum. Results of the protein expression by western blotting and ABCG2 mRNA expression were consistent with the immunohistochemical staining. This showed that ABCG2 mRNA expressions in hyperuricemia rats decreased significantly to 33.85% and 42.62% in jejunum and ileum respectively, and ABCG2 protein expressions by western blotting in them decreased markedly to 67.08% and 74.32% (Figs. 2 and Fig. 4) in hyperuricemia rats. This demonstrated that excessive intake of fructose would alter the mRNA and protein expressions of ABCG2 in the intestine. In order to determine the underlying molecular mechanisms of uricosuric effects of chicory, the mRNA and protein expressions of ABCG2 in jejunum and ileum responsible for intestinal uric acid excretion were examined. Notably, ABCG2 mRNA expression levels of the high and middle doses of chicory were increased to 231.34% and 383.17% in jejunum, and protein expression levels of them in jejunum were markedly enhanced to 153.98% and 199.33% by western blotting, when compared with hyperuricemia rats respectively. In ileum, only the high dose of chicory increased significantly to 256.22% and 169.16% in the expressions of ABCG2 mRNA and protein respectively when compared to the model group.

This is the first report that chicory has the ability of increasing mRNA and protein expression levels of ABCG2. These findings which are combined with our previous data illustrate that chicory has multiple effects on treating hyperuricemia, including the inhibition of uric acid production, and the excretion of uric acid via the kidney and the intestine, which embodies the characteristics of multi-target and multi-channel overall treatment of traditional Chinese medicine.

## Conclusion

In conclusion, chicory has the uricosuric effect in fructose-induced hyperuricemia rats, which was associated to accelerating intestinal excretion of uric acid by up-regulating the mRNA and protein expressions of ABCG2 in the intestine.

## Abbreviations

ABCG2: ATP-binding cassette transporter, sub-family G, member 2
BEN: Benzbromarone-treated group
CHI-H: High-dosage chicory treated group
CHI-L: Low-dosage chicory treated group
CHI-M: Middle-dosage chicory treated group
CON: Control group
IHC: Immunohistochemistry.
MOD: Model group
