# Correction to: *Cichorium intybus* L. promotes intestinal uric acid excretion by modulating ABCG2 in experimental hyperuricemia

**DOI:** 10.1186/s12986-021-00638-0

**Published:** 2021-12-27

**Authors:** Yu Wang, Zhijian Lin, Bing Zhang, Anzheng Nie, Meng Bian

**Affiliations:** Department of Clinical Chinese Pharmacy, School of Chinese Pharmacy, Beijing University of Chinese Medicine, Beijing, China

## Correction to: Nutrition & Metabolism (2017) 14:38 10.1186/s12986-017-0190-6

Following the publication of the original article [1], errors were identified in the Abstract, Tables 1 and 2, and Figure 3.

The sentence currently reads:

These findings indicate that chicory increases uric acid excretion by intestines, which may be related to the stimulation of intestinal uric acid excretion via down-regulating the mRNA and protein expressions of ABCG2.

The sentence should read:

These findings indicate that chicory increases uric acid excretion by intestines, which may be related to the stimulation of intestinal uric acid excretion via up-regulating the mRNA and protein expressions of ABCG2.

The original article [1] has been corrected.

**Table 1:** Body weight of rats during experimental days (n = 16, g)

Groups	0d	10d	20d	30d	40d
CON	251.31 ± 10.79	324.79 ± 14.79	358.12 ± 14.11	398.38 ± 21.24	422.49 ± 25.14
MOD	248.38 ± 11.02	311.45 ± 19.66	347.19 ± 22.97	387.68 ± 27.10	416.23 ± 32.75
BEN	243.45 ± 10.01	312.36 ± 11.58	344.04 ± 14.44	383.26 ± 18.52	405.41 ± 19.26
CHI-H	248.57 ± 11.19	316.72 ± 23.56	357.91 ± 27.60	397.21 ± 33.74	418.50 ± 40.09
CHI-M	247.94 ± 14.50	308.68 ± 22.28	347.85 ± 26.18	391.55 ± 32.40	412.93 ± 37.68
CHI-L	246.23 ± 12.16	315.35 ± 19.69	356.63 ± 22.61	395.39 ± 25.87	417.62 ± 30.46

**Table 2:** Uric acid-lowering effects of intragastric chicory in the hyperuricemic rats (n = 16, μmol/L)

Groups	0d	10d	20d	30d	40d
CON	51.47 ± 21.30	66.44 ± 26.12	76.22 ± 22.57	133.80 ± 33.23	74.10 ± 24.41
MOD	56.72 ± 28.76	87.63 ± 27.34*	98.07 ± 22.23**	177.08 ± 44.99**	95.80 ± 18.01**
BEN	50.56 ± 26.81	74.66 ± 35.29	75.64 ± 14.69^##^	147.11 ± 30.84^#^	82.60 ± 19.76
CHI-H	50.33 ± 21.91	52.40 ± 16.77^##^	66.17 ± 21.09^##^	145.61 ± 36.58^#^	73.82 ± 35.90^#^
CHI-M	53.08 ± 25.51	55.90 ± 29.62^##^	68.82 ± 16.84^##^	112.52 ± 45.48^##^	83.07 ± 41.07
CHI-L	50.22 ± 21.67	70.05 ± 32.18	65.09 ± 28.36^##^	155.27 ± 44.47	98.11 ± 9.46

*P < 0.05, **P < 0.01 versus the CON group; ^#^*P* < 0.05, ^##^*P* < 0.01 versus the MOD group. Means with different superscript lowercase letters in the same column are significantly different

**Fig. 3:**
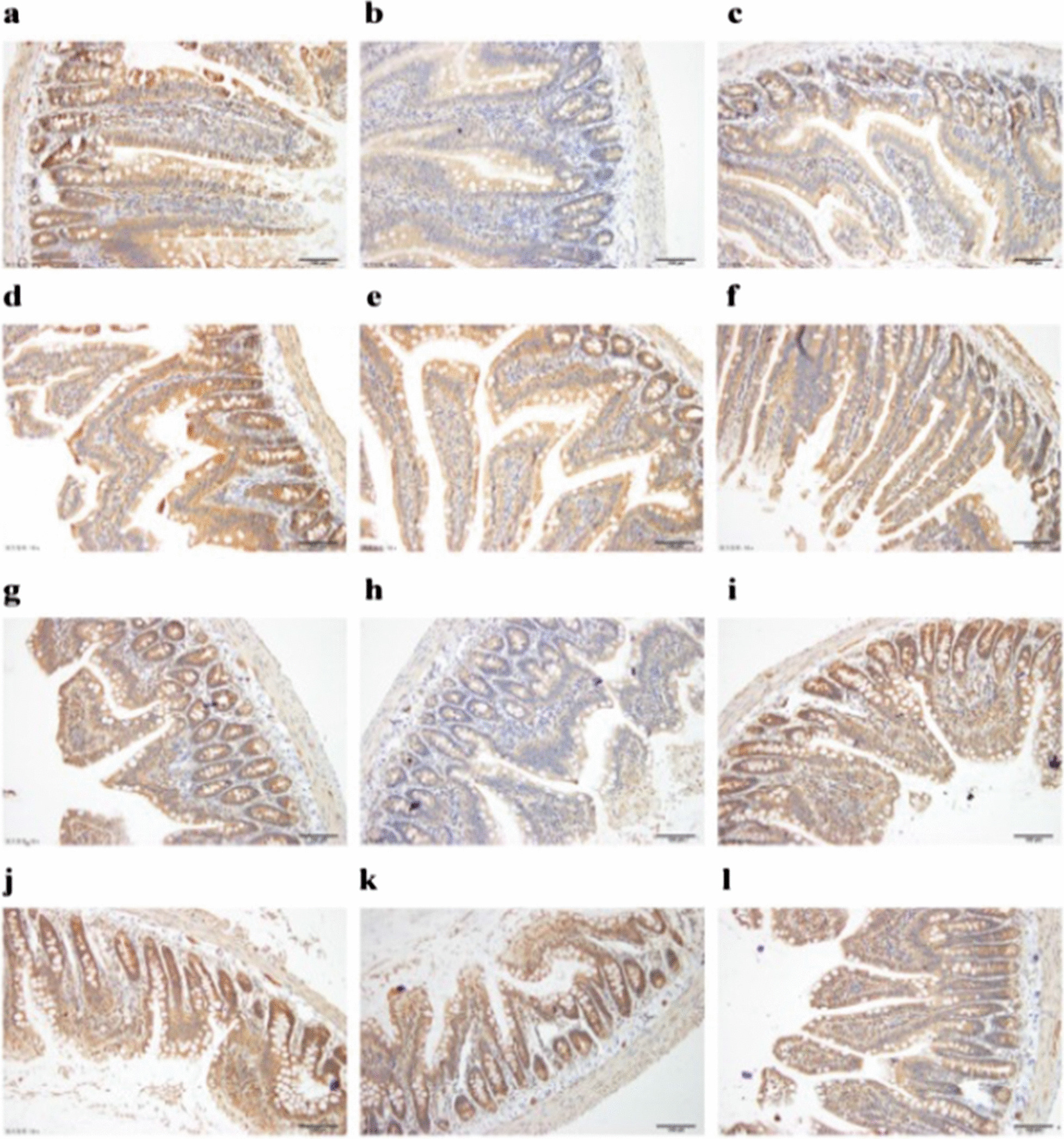
Location of ABCG2 and effects of chicory on ABCG2 protein expression. **a** Jejunum of normal rat ABCG2 IHC stain (×20 objective lens). **b** Jejunum of model rat ABCG2 IHC stain (×20 objective lens). **c** Jejunum of benzbromarone rat ABCG2 IHC stain (×20 objective lens). **d** Jejunum of chicory extract high-dose rat ABCG2 IHC stain (×20 objective lens). **e** Jejunum of chicory extract middle-dose rat ABCG2 IHC stain (×20 objective lens). **f** Jejunum of chicory extract low-dose rat ABCG2 IHC stain (×20 objective lens). **g** Ileum of normal rat ABCG2 IHC stain (×20 objective lens). **h** Ileum of model rat ABCG2 IHC stain (×20 objective lens). **i** Ileum of benzbromarone rat ABCG2 IHC stain (×20 objective lens). **j** Ileum of chicory extract high-dose rat ABCG2 IHC stain (×20 objective lens). **k** Ileum of chicory extract middle-dose rat ABCG2 IHC stain (×20 objective lens). **l** Ileum of chicory extract low-dose rat ABCG2 IHC stain (×20 objective lens). Low expression of model group in jejunum and ileum (*P* < 0.05, *P* < 0.01 vs. control group slices), which developed heavy stains; inhibition of ABCG2 by chicory (*P* < 0 .01, *P* < 0 .01 vs. model group slices)
